# The Foreign Journals: Medical Jurisprudence and Toxicology

**Published:** 1841-10

**Authors:** 


### MEDICAL JURISPRUDENCE AND TOXICOLOGY.

*Case of Death by Hanging, with numerous Wounds on the Person.* By Dr. Krügelstein, of Ohrdruff.

The deceased was about forty-eight years old; she had borne several children, and enjoyed good health and spirits up to the time of her last accouchement. She then became morose and unhappy, and attempted to drown herself. One morning she was found hanging, quite dead, in a cowshed adjoining her bedroom.

On examining the room in which she slept, some coagulated blood was found near the bed on the floor, and there were marks of bloody fingers on the walls, but there was no trace of blood on the bed itself. From this room to the shed in which her body was found there were no traces of blood, but there were impressions of bloody hands on the walls of the shed; and the beam from which her body was suspended as well as the cord was slightly stained. On the floor beneath the body there were marks of the fæces having been discharged. The roof of the shed was low, but sufficiently high to allow a person to be freely suspended from the floor. There was some dried blood on the face and dress.

The following appearances were met with:

On the tuberosity of the os occipitis, a severe contusion of a livid colour and about two inches in circumference. On cutting into it coagulated blood was found beneath but the skull was uninjured. Over the left temple there were seventeen incised wounds about two or three inches long, some penetrating to the bone, but none involving the temporal artery. On the left side of the os occipitis were two superficial cuts, and on the upper part of the right and left parietal bones were many others of a similar character. On the right temple there were so many deep incisions as to have completely destroyed the temporal muscle. Over the sagittal suture was a long wound penetrating to the bones.

The face was pale and free from any marks of violence; the eyelids were closed, and the eyes not projecting. The mouth was open; the tongue not prominent; there was no appearance of frothy mucus either in the mouth or nose.

Around the neck and above the larynx a tolerably deep depression had been produced by the cord. This extended beneath the angle of the jaw and the mastoid process on either side to the nape of the neck. Notwithstanding the cord had thus caused a deep and well-defined mark, there was no ecchymosis, if we except a slight bluish tint on the left side; nor had the skin acquired that parchment-like character which is often seen in the hanged. Neither the os hyoides, trachea, nor the vertebræ of the neck had sustained any mechanical injury.

The surface of the body was more or less covered with blood which had flowed from the wounds. Over the sternum and region of the stomach numerous but small incised wounds were perceived, which, from their appearance, had evidently been produced several days before those met with on the head. This was proved by the fact that some of them were passing into the suppurative stage.

On opening the cranium, splinters of the internal table were found corresponding to the wound in the region of the sagittal suture. These pressed on the dura mater, but had not injured it. There was no congestion, effusion, or injury to the brain, and the only morbid appearance was an hydatid cyst in one part of the pia mater. The lungs were of a bluish white colour, not particularly congested; a bloody froth escaped on making an incision into them. The heart and the abdominal viscera were in a normal state. The bladder was empty. The genitals were not turgid, as they are often described to be in females who have died by hanging.

The medico-legal questions to determine here were, as to whether: 1, the numerous wounds on the body had been self-inflicted; 2, whether the hanging was the result of suicide or murder.

The account given of the deceased showed that she had a predisposition to suicide. This is borne out medically by the discovery of hydatids in the pia mater.

In the shed where the body of the deceased was hanging an axe was found with which she had no doubt produced the injuries to her head. The edge of the axe was sufficiently sharp to account for the production of the numerous incised wounds found in the scalp, and the weapon was long enough to permit her to have produced the *severe contusion found on the os occipitis.* Had such a weapon been used by a murderer, one or two wounds only, and those of a much more severe character would have been produced. As it was there were no less than *fifty-five* wounds about the scalp, and most of these unimportant.

As negative proofs of this not being a homicidal act, may be mentioned: 1, that there was no mark of violence on the person, discomposure of the dress, or disturbance of the furniture in the room; 2, the act must have been perpetrated at a time when any noise or struggling would have been heard by the neighbours; but nothing of the kind was remarked.

The conclusions of the inspectors in this case were:

1. That the deceased did not die from the wounds found on her person nor from the loss of blood.
2. That death was the result of hanging, operating partly through pressure on the nerves and partly through suffocation.
3. That it was an act of suicide.

[REMARKS. This case is in many points interesting to the medical jurist. Had it occurred in England, it is scarcely necessary to observe that a coroner's jury would, under the circumstances, have deemed an inspection of the body useless, and as only giving rise to unnecessary delay and expense. Yet it is by the accurate observation of cases of this kind that materials can be collected, which will enable a medical witness to prevent coroners' juries from falling into mistakes in future investigations where the circumstances are of a more doubtful character. Out of the hundreds of cases of suicidal hanging which occur in this country, how many bodies are inspected by the order of a coroner? Scarcely one per cent.! We do not believe that so complete and accurate an investigation of the subject of hanging could be produced from the records of coroners' inquests, although the opportunities for benefiting science by this kind of information have been numberless.

This case possesses more than usual interest: 1, On account of the great number of wounds found on the head; 2, from the *nature* of some of the wounds, thus those over the sagittal suture were sufficient to cause a separation of the inner table of the skull; 3, from the situation. One violent contusion was seen on the os occipitis, a most unusual situation for a suicidal injury. It is remarkable that from the two last-mentioned circumstances concussion of the brain, depriving the person of the power of subsequently hanging herself, had not resulted, since there was evidently violence enough to account for an immediate loss of sense and motion. On the whole, suicide seems to have been more established from *general* than from *medical* facts; and had the deceased been found thus suspended in an exposed place, a strong suspicion of murder might fairly have arisen in the minds of the inspectors.]

*Henke Zeitschrift für die Staatsarzneikunde.* i. 1840.

---

*Poisoning by a Tobacco Enema.* B. M. Tavignot.

A strong man aged fifty-five, who had laboured for some time under dysuria from enlarged prostate, and more recently suffered from the presence of ascarides in the rectum, was subjected to the action of a tobacco injection, made by a decoction of 12 grs. of the tobacco in 6 oz. of water. Seven or eight minutes afterwards, the patient appeared in a slight stupor, with cephalalgia, and unusual paleness of the face. He complained of pain in the abdomen, and his answers to questions were troubled. Two purgative injections were successively administered. A stimulant potion was given; sinapisms applied to the inferior extremities, and blood taken to the amount of three *palettes.* Paleness became more and more marked, respiration more and more laboured, stupor, intelligence altogether lost; convulsive movements of the arms, then of the legs, and afterwards of the whole body, which progressively augmented during six or seven minutes, and were succeeded by extreme prostration. The patient fell into a comatose state and died.

*Revue Médicale. Décembre,* 1840.

---

*Medico-legal Question on the Criminal Production of Abortion in case of an Acephalous Monster.*

On the 2d of June last at the Assizes at Drôme a girl was accused of procuring abortion by criminal means. The fœtus when it came into the world was acephalous; there was no vertebral canal to the spinal column; the spinal marrow and nerves were in a rudimentary state and immediately under the skin. One of the lungs was but half the size of the other, and both sunk in water The forehead was completely absent, the chin was almost confounded with the chest, and there was scarcely any neck. The fœtus was of about six months. It was proved by MM. Morin and Biguel that it had never respired, and that its life ceased with gestation; but they also remarked on its head a wound and a small ecchymosis which they regarded as a probable result of a criminal manœuvre, executed by the aid of a pointed instrument shortly before abortion, and they thought that the death of the fœtus was the consequence, whilst in the womb of the mother.

The most important question which presented itself, and which was seized by the counsel for the prisoner was, in a case where the material fact of abortion provoked by the accused was demonstrated, could the penalty apply for delivering herself of a monstrous fœtus, incapable of life, and could a deed be considered as a crime which had caused no prejudice to society nor to an individual? (individualité.) The defender after having distinguished abortion from infanticide, which cannot be a crime when the infant is not viable; and alluded to the remarkable fact peculiar to abortion, that the law does not punish the intention, even when it is manifested by the commencement of execution, argued that it is not the endeavour that the law punishes, but the injurious deed alone. "The destruction of a monster incapable of life cannot injure it. The fact, then, cannot constitute the crime of abortion. If it be the deed prejudicial to generation that the law arraigns, it must be said that in this case there existed no prejudice, no crime, and no penalty could be awarded." The jury acquitted the prisoner, notwithstanding the opposition of the *ministre public.*

*Gazette Médicale de Paris.* 3 *Juillet*, 1841.

